# Structural Evidence for the Substrate Channeling of Rice Allene Oxide Cyclase in Biologically Analogous Nazarov Reaction

**DOI:** 10.3389/fchem.2018.00500

**Published:** 2018-10-30

**Authors:** Sereyvath Yoeun, Kyoungwon Cho, Oksoo Han

**Affiliations:** ^1^Department of Molecular Biotechnology and Kumho Life Science Laboratory, College of Agriculture and Life Sciences, Chonnam National University, Gwangju, South Korea; ^2^Faculty of Chemical and Food Engineering, Institute of Technology of Cambodia, Phnom Penh, Cambodia

**Keywords:** allene oxide cyclase, rice, nazarov reaction, octadecanoid, oxylipin, jasmonic acid

## Abstract

Allene oxide cyclase (AOC) is a key enzyme in the jasmonic acid (JA) biosynthetic pathway in plants, during which it catalyzes stereospecific conversion of 12,13(*S*)-epoxy-9(*Z*),11,15(*Z*)-octadecatrienoic acid (12,13-EOT) to *cis*(+)-12-oxophytodienoic acid. Here, rice allene oxide cyclase (OsAOC) was localized to the chloroplast and its native oligomeric structure was analyzed by gel electrophoresis in the absence and presence of a protein-crosslinking reagent. The results suggest that OsAOC exists in solution as a mixture of monomers, dimers, and higher order multimers. OsAOC preferentially exists as dimer at room temperature, but it undergoes temperature-dependent partial denaturation in the presence of SDS. A heteromeric 2:1 complex of OsAOC and rice allene oxide synthase-1 (OsAOS1) was detected after cross-linking. The yield of *cis*(+)-12-oxophytodienoic acid reached maximal saturation at a 5:1 molar ratio of OsAOC to OsAOS1, when OsAOC and OsAOS1 reactions were coupled. These results suggest that the OsAOC dimer may facilitate its interaction with OsAOS1, and that the heteromeric 2:1 complex may promote efficient channeling of the unstable allene oxide intermediate during catalysis. In addition, conceptual similarities between the reaction catalyzed by AOC and Nazarov cyclization are discussed.

## Introduction

**Figure 1:**
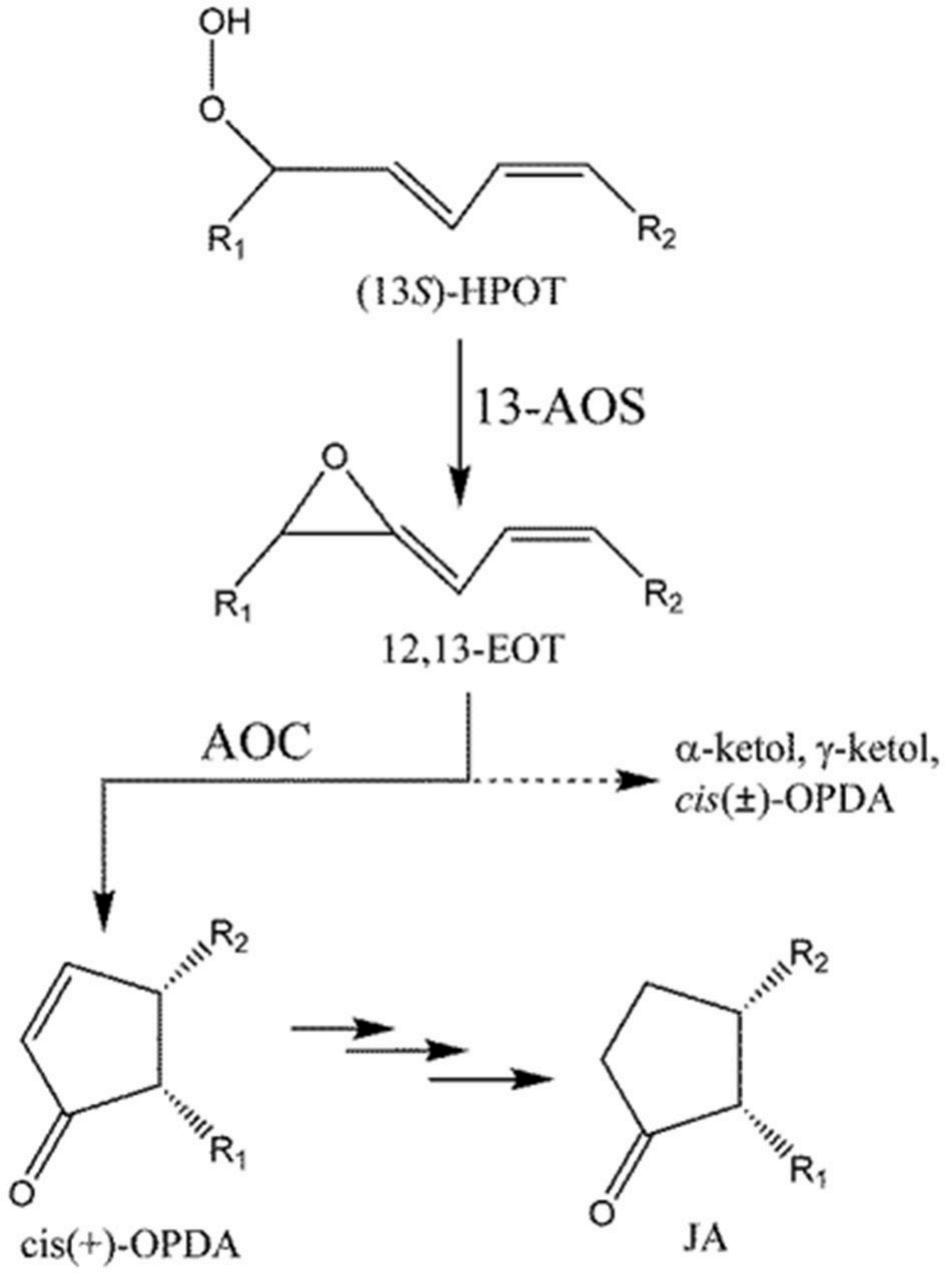
Formation of *cis*-OPDA by AOS and AOC. Dotted line (- - - - - -) indicates a non-enzymatic process that occurs in the absence of AOC. R_1_ = C_5_H_9_, R_2_ = C_7_H_14_COOH.

Biosynthesis of phyto-oxlipins is initiated by lipoxygenase (LOX), which converts polyunsaturated fatty acids (PUFAs) into their corresponding hydroperoxides. Hydroperoxyoctadecatrienoic acid (HPOT), synthesized from α-linolenic acid (LnA), is further metabolized to *cis*(+)-12-oxophytodienoic acid (*cis*(+)-OPDA) by consecutive reactions catalyzed by allene oxide synthase (AOS) and allene oxide cyclase (AOC) (Figure 1; Blée, 2002; Mosblech et al., 2009; De León et al., 2015).

AOS and AOC localize to the chloroplast (Wasternack, 2007). *cis*(+)-OPDA, generated in the chloroplast, is then transferred to the peroxisome, where it is reduced to 3-oxo-2(2′*Z*-pentenyl)-cyclopentane-1-octanoic acid (OPC-8:0) by OPDA reductase (OPR), and finally converted into jasmonic acid (JA) by three cycles of β-oxidation (Schaller et al., 2000). Jasmonates including OPDA play important functions in plant defense (Sharma and Laxmi, 2016) and have been proposed as important therapeutic (anti-cancer) or insect-repellent compounds (Raviv et al., 2013). OPDA, structurally similar to common cyclopenta(e)none compounds, plays important roles in development and stress response pathways in nonvascular plants, such as moss and liverwort, where JA is not found (Stumpe et al., 2010; Yamamoto et al., 2015). More importantly, OPDA plays a distinct role from JA in flowering plants. For example, both OPDA and JA are induced by wounding, but only OPDA is induced by drought (Savchenko et al., 2014). Global transcriptome analysis identified 172 wound-response genes in *Arabidopsis thaliana* whose expression is regulated by OPDA but not JA (Taki et al., 2005). In A. thaliana, OPDA also plays a key role in susceptibility to infection by root-knot nematodes (Gleason et al., 2016). These results indicate that, at least in some species, OPDA plays unique roles in stress responses in plants, in addition to its importance as an intermediate in JA biosynthesis.

Rice genomes encode at least 16 LOXs, 5 AOSs, and 1 AOCs which almost identical to each other (Agrawal et al., 2004). LOX and AOS exhibit diverse substrate specificity and relatively broad regiospecificity. For example, LOX can utilize either linoleic (18:2) or linolenic (18:3) acid as a substrate to produce 9- or 13-positional isomeric hydroperoxide, which is converted into the corresponding allene oxide by AOS. However, AOC utilizes only 12,13(*S*)-epoxy-9(*Z*),11,15(*Z*)-octadecatrienoic acid (12,13-EOT) produced from (13*S*)-HPOT and stereospecifically produces *cis*(+)-OPDA as the only enantiomeric product (Figure 1; Hofmann and Pollmann, 2008; Mosblech et al., 2009). Therefore, AOC demonstrates high substrate-and stereo-specificity and this specificity is crucial because the stereospecific cyclization of 12,13-EOT imposes the chirality at C-9 and C-13 of the cyclopentenone structure in OPDA, which in turn determines the enantiomeric configuration of JA. The unstable allene oxide substrate, 12,13-EOT, can be non-enzymatically hydrolyzed into α- and γ-ketols in aqueous media, or alternatively, in the absence of AOC, it can undergo non-enyzmatic cyclization, forming racemic *cis*(±)-OPDA (dotted line; Figure 1). The stereospecific cyclization of 12,13-EOT in the presence of AOC is mechanistically analogous to the stereoselective elctrocyclization of divinyl ketone in the Nazarov reaction, because stereochemical control is governed by the principles of pericyclic ring closure and because of the shared cyclopentenone structure of the reaction products (Hofmann and Pollmann, 2008; Tius, 2014; Figure S1). Therefore, the chemistry of the AOC reaction can be considered a biological analog of Nazarov cyclization in organic chemistry.

The oligomeric structure of AOC may influence its activities and the rate of flux through the JA biosynthetic pathway. Crystallographic studies revealed that AOC exists as a trimer (Hofmann et al., 2006; Neumann et al., 2012) in the crystalline state, while AOC from dried corn seeds was reported to exist predominantly as a dimer in solution (Ziegler et al., 1997). In this study, the oligomeric structure of OsAOC in solution was analyzed by gel electrophoresis in the presence of chemical protein cross-linking reagent BS^3^. Oligomeric structures of partially denatured OsAOC and an OsAOC-OsAOS1 fusion protein were also investigated. The role of dimeric OsAOC in substrate channeling and the conceptual similarity between the reaction catalyzed by AOC and Nazarov cyclization are discussed.

## Materials and methods

### Expression and purification of proteins

Full-length OsAOC (FOsAOC, acession no. AJ493664) containing the localization sequence and truncated OsAOC (tOsAOC) were cloned into the pRSETB vector (Invitrogen). To improve expression and protein stability, the OsAOC coding region was fused with two different leading/linker sequences in between the 6 × histidine epitope tag (6 × His-tag) and the N-terminus of OsAOC, as shown in Figure 3A. The resulting plasmid constructs were transformed into *E. coli* strain BL21(DE3) pLysS. Single-transformant derived colonies were inoculated into LB medium, grown at 37°C with shaking (200 rpm) to an optical density (OD_600_) of 0.4–0.6, and then induced by addition of isopropyl-β-D-thiogalactopyranoside (IPTG) to a final concentration of 0.2 mM. After 7 h, bacterial cells were harvested by centrifugation. The cells were resuspended in lysis buffer (50 mM sodium phosphate, 0.2 mM PMSF, pH 7.5) and sonicated. The soluble protein was precipitated by ammonium sulfate with increasing saturated percentage from 20 to 50%. Salt was removed from fraction containing the recombinant OsAOCs by dialysis. The recombinant proteins were purified by ion exchange chromatography using a Q-sepharose column (Sigma) in buffer containing 5 mM EDTA, 50 mM sodium phosphate (pH 7.5). Optionally, the cells were resuspended in lysis buffer (50 mM sodium phospahte, 10 mM imidazole, 250 mM NaCl, 0.2 mM PMSF, pH 8.0), sonicated, and the recombinant OsAOCs were purified by Nickel column (ProBond, Invitrogen) in the absence of EDTA. The column was eluted with buffer containing imidazole according to the method provided by supplier. If necessary, EDTA was added to the eluted fractions with final concentration of 5 mM to prevent the cleavage of TP. For cross-linking experiments, EDTA and imidazole were removed by centrifugal buffer exchange (Pall Corporation, 10K). The biochemically relevant OsAOC prepared from OsAOC(I) in the absence of EDTA was used to characterize biochemical properties. Rice allene oxide synthase-1 (OsAOS1, acession no. AY055775) was employed for coupled reaction of OsAOC as reported previously (Yoeun et al., 2013).

Truncated OsAOC (tOsAOC) in Figure 3 and full sequence OsAOS1 and were fused in two different arrangements, as shown in Figure S5. OsAOC-OsAOS1 contained 13 amino acids (EFKDPSSRSAAGT) as a linker connecting two genes and 6 × His-tag at N- and C-termini; whereas the OsAOS1-OsAOC fusion construct did not use a linker in between the two open reading frames. The OsAOC-OsAOS1 plasmid was constructed directly by employing the NheI restriction site. The predicted lengths of the two fusion proteins are 702 and 668 amino acids with estimated molecular mass of 77.3 and 73.4 kDa for OsAOC-OsAOS1 and OsAOS1-OsAOC, respectively. Both constructs were inserted into pET-28b expression vector (Novagen) and transformed in *E. coli* strain BL21(DE3). Single colonies were inoculated into LB medium and grown at 37°C, 200 rpm to an optical density (OD_600_) of 0.4–0.6. The fusion proteins were then induced by the addition of IPTG at a final concentration of 1 mM and grown for 12 h at 25°C with shaking (200 rpm). Bacterial cells were harvested by centrifugation and lysed by sonication in buffer A (50 mM sodium phosphate, 0.2 mM PMSF, 0.1% Emulphogene, 250 mM NaCl, 10 mM Imidazole, pH 8.0) or buffer B (50 mM sodium phosphate, 0.2 mM PMSF, 0.1% Emulphogene, 5 mM EDTA, pH 7.5) for OsAOC-OsAOS1 or OsAOS1-OsAOC, respectively. OsAOC-OsAOS1 was purified by nickel column (ProBond, Invitrogen) and OsAOS1-OsAOC was purified by Q-sepharose column (Sigma).

### Gel electrophoresis and edman sequencing

SDS-PAGE and native gel electrophoresis were conducted using a protocol reported previously (Bollag et al., 1996) with 15 or 6–15% gradient gel for SDS-PAGE. Protein samples were prepared with 0.4% SDS for denaturing or without SDS for native gel electrophoresis. N-terminal sequencing of OsAOC was performed by Life Science Laboratories (Seoul, Korea). Approximately 5 μg of protein was loaded and separated on a 15% acrylamide gel for SDS-PAGE. Then, protein was transferred to a PVDF membrane (0.45 μm) using a Sigma semi-dry blotter following the supplier's protocol. The membrane was briefly washed with distilled water and stained with Coomassie brilliant blue R-250. The peptide samples were prepared by the pulsed liquid PVDF protein method as described by the manufacturer (Applied Biosystems, USA). Samples were injected onto the PTH column for HPLC and detected at 269 nm using Procise software with a gradient of solvent A (3.5% tetrahydrofuran in water) and solvent B (12% isopropanol in acetonitrile) at a flow rate of 325 μL per min in a LC 492 Protein Sequencing System (Applied Biosystems, USA).

### Immunofluorescence microscopy

Rice leaves (*Oryza sativa* L. Nakdong) were harvested 14 days after germination. The leaf segments were fixed in 5% acetic acid, 10% formalin, 80% ethanol in distilled water, and gradually dehydrated by sequential incubation in 80, 90, 95%, and pure ethanol. Images were obtained by confocal immunofluorescence microscopy using anti-AOC rabbit antiserum (1:100), prepared as previously reported (Yoeun et al., 2015).

### OsAOS1-OsAOC assay

(13*S*)-HPOT was prepared from 0.5 mM linolenic acid (LnA) using 7 μg soybean LOX (L7395, Sigma-Aldrich) in 1 mL 50 mM sodium phosphate buffer (pH 7.0) for 10 min at room temperature. An assay of the coupled OsAOC-OsAOS1 reaction was conducted as follows. OsAOS1 and OsAOC(I) (total 5 μg combined) were mixed to achieve molar ratios from 1:0 to 1:20 and added to (13*S*)-HPOT prepared by soybean LOX, to reach a total reaction volume of 2 mL. Reaction products were extracted. *cis*-OPDA was separated by straight phase HPLC (SP-HPLC), and the stereochemistry of *cis*-OPDA was analyzed by chiral phase HPLC (CP-HPLC) as described previously (Yoeun et al., 2013). Alternatively, the reaction mixture was injection directly, without extraction onto reverse phase HPLC (RP-HPLC), and HPLC was developed in methanol: water: acetic acid (80:20:0.01). All HPLC analyses were performed with UV detection at 205, 220, and 234 nm for α-ketol, OPDA, and HPOT, respectively.

### Chemical cross-linking and western blot

The purified OsAOC(I) and OsAOS1 were cross-linked with BS^3^ [bis(sulfosuccinimidyl)suberate] as described previously (Yoeun et al., 2015). For cross-linking, OsAOC(I) and OsAOS1 were mixed at a molar ratio of 2:1 and the proteins were precipitated by 50% saturated ammonium sulfate. The precipitated proteins were resuspended in sodium phosphate buffer and crosslinked. Approximately 2.5 μg total protein was analyzed by 6–15% gradient SDS-PAGE. The purified OsAOC(I) and OsAOS1 were used to immunize rabbits and their polyclonal antisera were obtained by Anygen Co., Ltd. (Gwangju, Koroea) for immunoblotting experiments as reported previously (Yoeun et al., 2015).

### Gel filtration

The oligomeric state of OsAOC was analyzed by gel filtration HPLC using a Bio-Silect SEC 250-5 column (7.8 × 300 mm, Bio-Rad). Approximately 30 μg recombinant OsAOC was loaded onto the column and eluted in 0.1 M sodium phosphate (pH 6.5), 0.2 M sodium chloride at 1 mL/min with detection at 280 nm. The molecular weight (MW) standards, β-amylase (200 kDa), BSA (66 kDa), ovalumin (44 kDa), carbonic anhydrogenase (29 kDa), and cytochrome c (12.4 kDa), were purchased from Sigma-Aldrich (USA).

## Results

### Chloroplast targeting peptide and OsAOC subcellular localization

**Figure 2:**
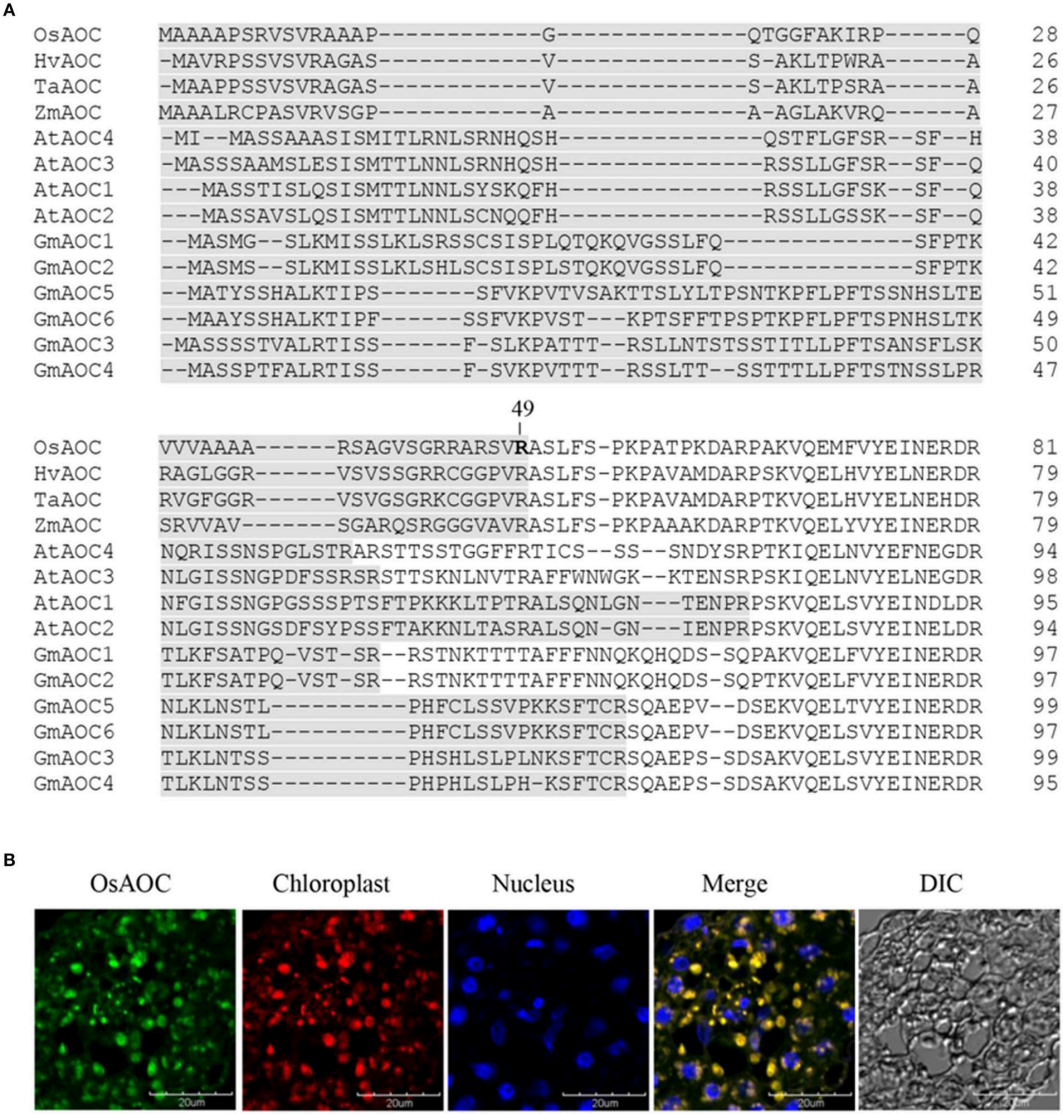
Chloroplast targeting sequences of AOC in plant species and cellular localization of OsAOC in rice leaves. **(A)** Comparison of chloroplast targeting sequences (gray) of monocot and dicot AOCs. Accession numbers: OsAOC (AJ493664), HvAOC (AJ308488), TaAOC (KF039887), ZmAOC (AY488136), AtAOC1 (AJ308483), AtAOC2 (AJ308484), AtAOC3 (AJ308485), AtAOC4 (AJ308486), GmAOC1 (HM803106), GmAOC2 (HM803107), GmAOC3 (HM803108), GmAOC4 (HM803109), GmAOC5 (HM803110), GmAOC6 (HM803111). **(B)** Immunofluorescence confocal microscopy was used to localize OsAOC in rice leaves. The green immunofluorescent signal corresponds to OsAOC. The red fluorescent signal is chlorophyll autofluorescence in the chloroplast. Blue fluorescence is nuclear. DIC indicates a phase contrast image of the microscopic field.

Several software programs were used to search the amino acid sequence of OsAOC (acession no. AJ493664) for putative chloroplast targeting motifs, as reported previously (Agrawal et al., 2003). The results showed a strong consensus, predicting that amino acids 1–49 of OsAOC are a functional chloroplast targeting peptide. AOC from monocot plants, including rice (OsAOC), barley (HvAOC), wheat (TaAOC), and maize (ZmAOC), share a putative cleavage position immediately after Arg-49 in OsAOC (Figure 2A), which would release the N-terminal chloroplast transit peptide (TP). The chloroplast localization of OsAOC was investigated by confocal fluorescence microscopy using anti-OsAOC antibody. The OsAOC protein was visualized with green immunofluorescence. Figure 2B shows that the green fluorescence from OsAOC clearly overlaps with red chlorophyll auto-fluorescence found uniquely in the chloroplast. This result confirms that OsAOC is localized to the chloroplast in rice leaves (Figure 2B).

### Removal of pre-sequences and chloroplast TP in recombinant OsAOC in *E. coli*

**Figure 3:**
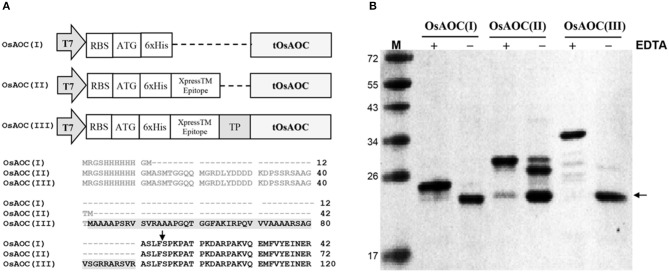
Construction of expression vectors and cleavage of pre-sequences in recombinant OsAOC. **(A)** Vector construction and N-terminal sequences of the three variants of OsAOC. OsAOC(I) and OsAOC(II) are the truncated OsAOC (tOsAOC), and OsAOC(III) is the full-length OsAOC (FOsAOC) containing targeting peptide (TP) indicated by gray shading. The predicted number of amino acid residues is 203, 233, and 281 in OsAOC(I), OsAOC(II), and OsAOC(III), respectively. Amino acid residues in gray are pre-sequences from the expression vector. **(B)** Inhibitory effect of EDTA on the excision of pre-sequences and TP in OsAOCs. Proteins were purified in buffer containing 5 mM EDTA to prevent self-cleavage during purification. EDTA was removed using centrifugal filters and samples were stored at 4°C for 1 week prior to analysis by SDS-PAGE. The + and – symbols indicate the presence or absence of EDTA in the sample. The arrow indicates the position of stable self-cleaved OsAOC.

Full-length OsAOC with TP (FOsAOC) and truncated OsAOC lacking TP (tOsAOC) were cloned into the expression vector pRSETB. A schematic diagram of three plasmid constructs and the corresponding variants of OsAOC protein is shown in Figure 3A. The OsAOC(I), (II), and (III) proteins were expressed in *E. coli* BL21(DE3) pLysS and detected by Western blot. The result shows that the recombinant OsAOC variants expressed from these plasmids were degraded to smaller protein fragments during purification (Figure 3B). Edman sequencing of purified OsAOC confirmed the absence of pre-sequences, indicating that they were excised (or removed by splicing) before or during purification, leaving an N-terminal serine residue that maps to coordinate 53, four amino acids from the predicted TP cleavage site (Figure 3A, arrow). Interestingly, the cleavage at Ser53 of OsAOC was inhibited by EDTA (Figure 3B). Proteins were purified in buffer containing 5 mM EDTA to prevent the cleavage during purification. EDTA was removed using centrifugal filters and samples were stored at 4°C for 1 week prior to analysis by SDS-PAGE. The + and – symbols in Figure 3B indicate the presence or absence of EDTA in the sample. The arrow indicates the position of the cleaved OsAOC.

### Effect of pre-sequence and TP on OsAOC activity and stereospecificity

**Figure 4:**
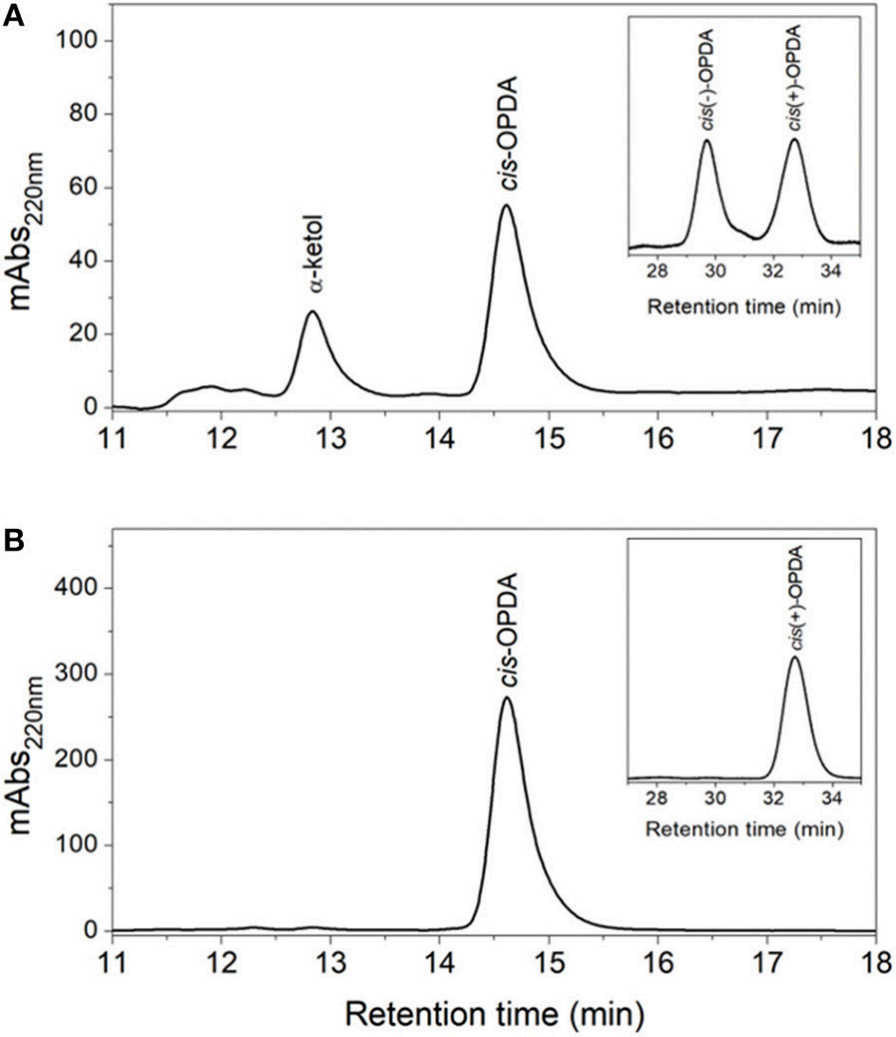
HPLC analysis of OsAOC(I) reaction products. **(A)** SP- and CP-HPLC analysis of non-enzymatic cyclization of 12,13-EOT. (13*S*)-HPOT was converted into 12,13-EOT by OsAOS1 and racemic *cis*-OPDA was produced by non-enzymatic cyclization. **(B)** SP- and CP-HPLC analysis of enzymatic cyclization of 12,13-EOT) into *cis*(+)-OPDA by the coupled reactions of OsAOC and OsAOS. Reaction products were extracted and loaded onto SP-HPLC. *cis*-OPDA-containing fractions were collected and analyzed by CP-HPLC (panel inserts).

The products of the coupled reactions of rice allene oxide synthase-1 (OsAOS1) and OsAOC(I) were extracted, separated by straight phase HPLC (SP-HPLC) and analyzed by GC-MS, as reported previously (Yoeun et al., 2013). The stereochemistry of the *cis*-OPDA reaction product was analyzed by chiral phase HPLC (CP-HPLC). Results are shown in Figure 4. Reverse phase HPLC (RP-HPLC) was employed to directly quantify reaction products without extraction (Figure S2), as necessary. *cis*-OPDA from nonenzymatic cyclization of 12,13-EOT (produced by OsAOS1) was a racemic mixture [*cis*(±)-OPDA] as expected (Figure 4A). In contrast, the coupled reactions of OsAOC(I) and OsAOS1 produced only the *cis*(+)-OPDA enantiomer via stereospecific cyclization of 12,13-EOT (Figure 4B). Furthermore, OsAOC(II), OsAOC(III), and the cleaved OsAOC (arrowed in Figure 3B) also catalyzed a stereospecific reaction (data not shown). The result showed to produce *cis*(+)-OPDA with similar efficiency as OsAOC(I), suggesting that the presence or absence of a pre-sequence including TP does not influence the activity or stereospecificity of OsAOC.

### Oligomeric state of OsAOC

**Figure 5:**
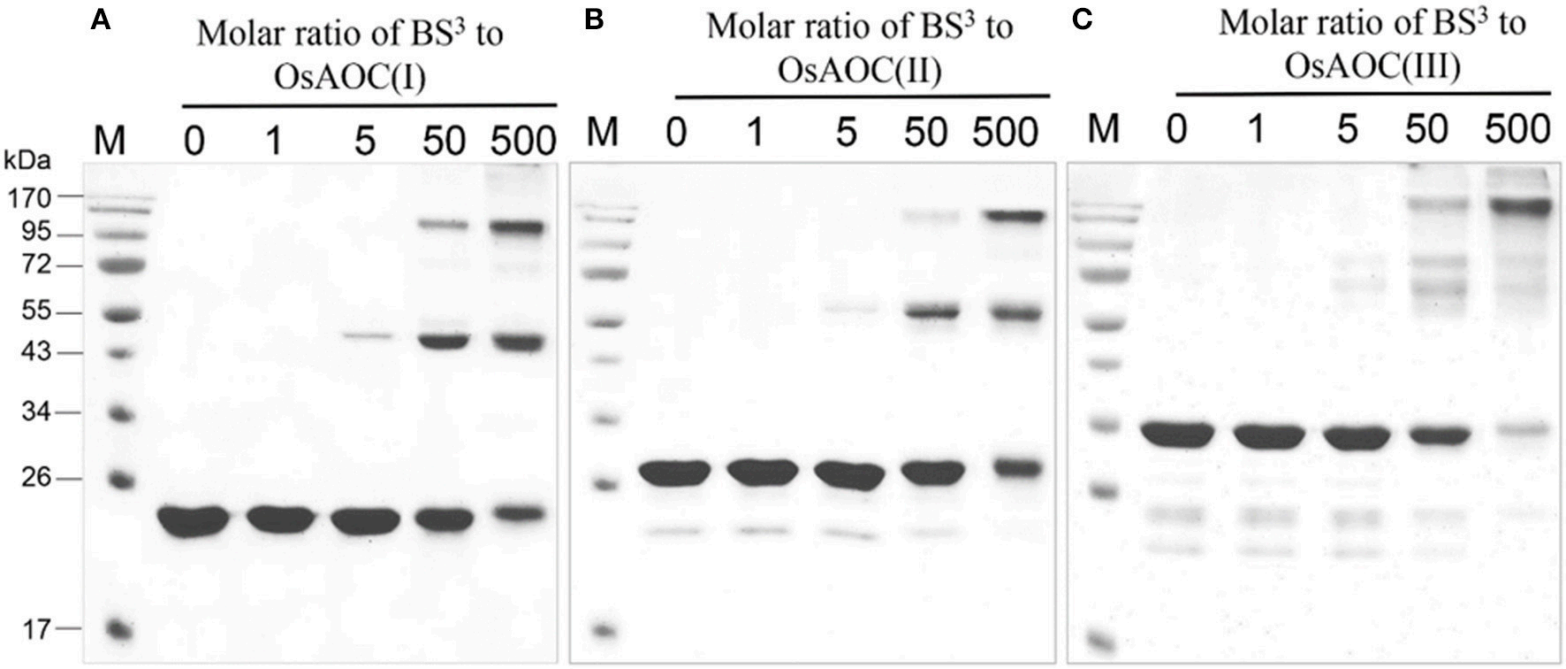
Oligomeric diversity of OsAOC in solution analyzed by protein cross-linking. **(A)** OsAOC(I), **(B)** OsAOC(II), **(C)** OsAOC(III). From left to right, samples contain an increasing molar ratio (from 0 to 500) of BS^3^ to recombinant OsAOCs ~5 μM, as indicated. Each reaction contained ≈2.5 μg recombinant protein. Reaction products were analyzed by 15% SDS-PAGE.

The oligomeric conformation(s) of OsAOC were examined by several methods. Native gel electrophoresis (Figure S3A) revealed that three recombinant OsAOC variants have distinct electrophoretic mobilities. OsAOC(I) and OsAOC(II), which lack the TP, exhibited a molecular mass of 146–480 kDa during electrophoresis under native conditions. In contrast, full length OsAOC(III), which retained the TP, did not enter the gel matrix. Multimeric forms of the recombinant OsAOCs [OsAOC(I) and OsAOC(III)] were also detected by gel filtration-HPLC (Figure S3B). The OsAOCs were detected in early-eluting fractions, indicating a high molecular weight multimeric form of OsAOC. Oligomeric structures of OsAOC were further characterized in detail after cross-linking with BS^3^ (11.4 Å) followed by SDS-PAGE (Figure 5). Diverse oligomeric structures were observed, including monomer, dimer, and higher multimers, depending the ratio of OsAOC to cross-linker. Dimeric forms began to appear at a 1:5 molar ratio of protein to cross-linker and higher multimers increased in abundance with increasing ratio of cross-linker to protein, with similar results for all three variants of OsAOC. However, higher multimers of OsAOC(III), which retains the TP, were more abundant than, higher multimers of OsAOC(I), which lacks the TP. In order to exclude nonspecific cross-linking, proteins were diluted 100-fold while maintaining the concentration of BS^3^, prior to analysis by Western blot (Figure S4).

**Figure 6:**
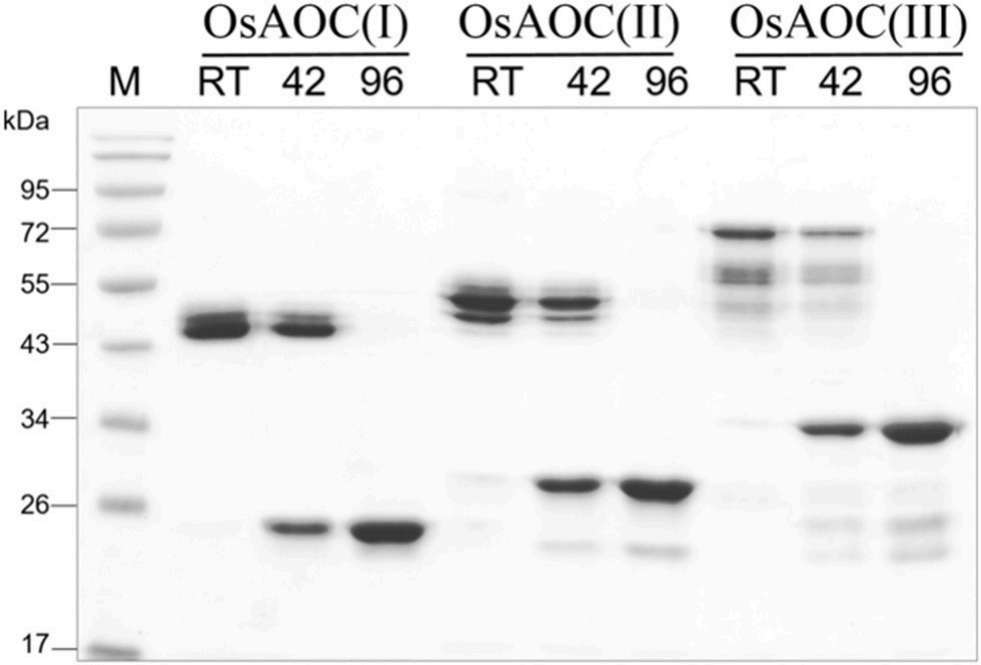
Analysis of the oligomeric structure of partially denatured OsAOCs. The each of three recombinant OsAOCs, such as OsAOC(I), (II), and (III) (~2.5 μg) was mixed with loading buffer (0.4% SDS), incubated for 5 min at 25°C (RT), 42, or 96°C, and immediately analyzed by SDS-PAGE (15% gel) using buffer containing 1% SDS.

The oligomeric structure of partially-denatured OsAOC and its dependence on temperature were analyzed in the presence of 0.4% SDS. Results are shown in Figure 6. Monomeric OsAOC was detected under fully-denaturing conditions of 0.4% SDS and 96°C as expected. Interestingly, only OsAOC dimers were observed at 25°C and both dimeric and monomeric forms were observed at 42°C in the presence of 0.4% SDS in all OsAOC variants. The results also indicate that partially denatured OsAOC exists primarily as a dimer at room temperature.

### Interaction between OsAOC and OsAOS1

**Figure 7:**
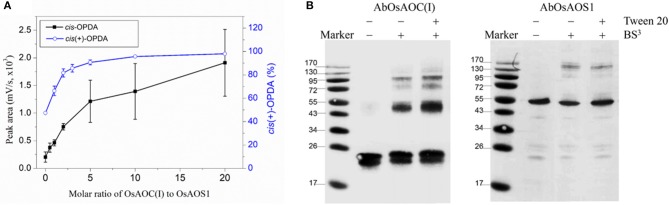
Possible interaction between OsAOC and OsAOS1. **(A)** Yield of OPDA with increasing molar ratios of OsAOC(I) to OsAOS1. Reaction mixtures at different molar ratios of OsAOC(I) to OsAOS1 were injected without extraction onto RP-HPLC and analyzed directly (see Figure S2). *cis*-OPDA abundance was determined from peak area-under-the-curve in the RP-HPLC chromatogram (*n* = 3). The ratio of *cis*(+)-OPDA to total *cis*-OPDA was determined from CP-HPLC (see insert to Figure 4). **(B)** Western blot analysis of the multimers formed between OsAOC(I) and OsAOS1 (0.04 μg/μl) in the presence of BS^3^ (1 mM) for 30 min at room temperature. SDS-PAGE sample buffer was added to each sample, which were boiled for 5 min and analyzed by Western blot using Anti-OsAOC and Anti-OsAOS1 antibodies (see Materials and Methods).

**Figure 8:**
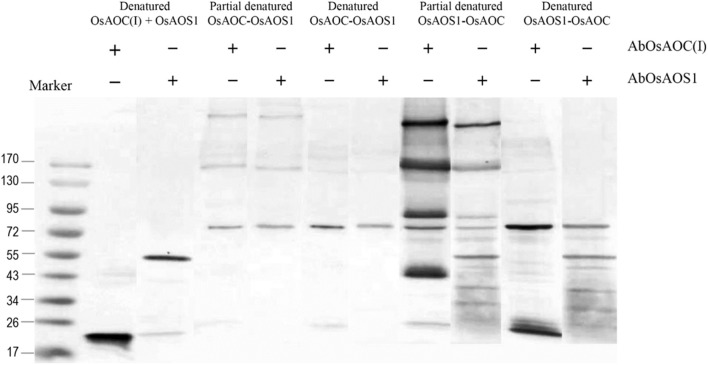
Analysis of the oligomeric structure of partially denatured fusion proteins of OsAOC and OsAOS1. Western blot analysis of partially denatured fusion proteins of tOsAOC and OsAOS1. Fusion proteins were mixed with loading buffer (0.4% SDS), incubated for 5 min at 25°C (RT) and analyzed on a 6–15% gradient acrylamide gel (6–15%) in the presence of SDS. Western blot using anti-OsAOS1 or anti-OsAOC was carried out as described.

Because allene oxides have a short half-life in aqueous media, the AOS and AOC reactions might be temporally and/or spatially coupled, as a mechanism to channel the unstable allene oxide forward in the JA biosynthetic pathway and prevent its premature dissolution. Therefore, in order to investigate possible interactions between OsAOC and OsAOS1, the stereospecificity and yield of catalysis by OsAOC(I) was first examined at a variable molar ratio of OsAOC(I) to OsAOS1. The results show that yield of *cis*(+)-OPDA increased hyperbolically with increasing molar excess of OsAOC(I) to OsAOS1, reaching saturation at an approximate ratio of 5:1 [OsAOC(I):OsAOS1; Figure 7A]. This is consistent with the idea that OsAOS1 and OsAOC facilitate efficient channeling of the allene oxide intermediate 12,13-EOT. Additional support for this possibility comes from our evidence that OsAOC(I) and OsAOS1 form a heteromeric protein complex after co-incubation in the presence of BS^3^. Western blot analysis indicated a band corresponding to molecular weight ≈100 kDa, which could tentatively be assigned as a 2:1 heteromeric complex of OsAOC(I) and OsAOS1 (Figure 7B). To investigate the putative interaction between OsAOC and OsAOS1, fusion proteins of OsAOC and OsAOS1 were designed as shown in Figure S5, and their oligomeric structures were analyzed under denaturing and partially-denaturing conditions. Unfortunately, the OsAOC-OsAOS1 fusion protein was not active (data not shown). This could indicate that the co-linear arrangement of OsAOC N-terminal to OsAOS1 does not support formation of an active protein conformation. However, under partially-denaturing conditions, an OsAOS1-OsAOC fusion protein did assume a mixed oligomeric state, which included monomers, dimers and other multimers (Figure 8). The OsAOS1-OsAOC fusion protein was readily spliced into OsAOS1 and OsAOC monomers, as expected based on previous results (Figure 3).

### Structural modeling of OsAOC

**Figure 9:**
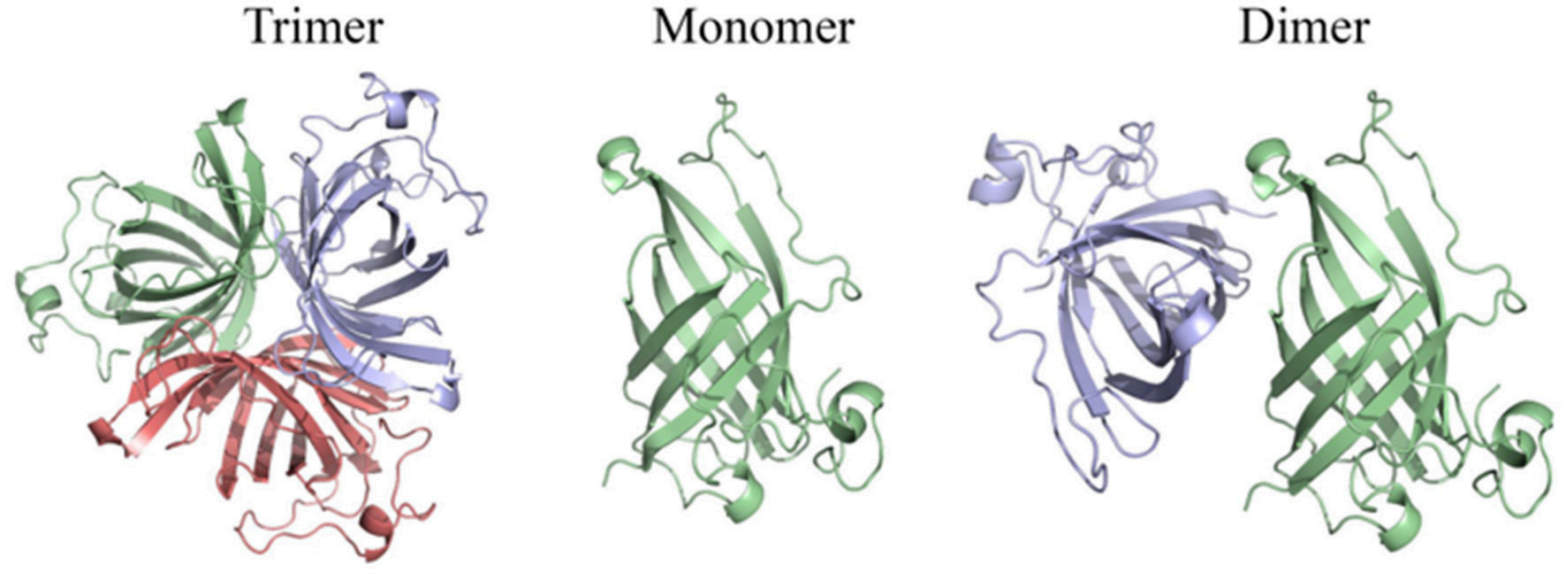
Homo-oligomeric structural models of OsAOC predicted by Galaxy Gemini tool (Lee et al., 2013) based on monomeric OsAOC.

The above results suggest that OsAOC exists as a mixture of monomers, dimers and higher multimers in solution. To gain insight into the subunit conformation and arrangement in these multimers, bioinformatic tools were used to generate a structural model for OsAOC mixed multimers. First, SWISS-Model (Biasini et al., 2014) was used to automatically model OsAOC with *Arabidopsis thaliana* AOC2 as a template. The algorithm predicted that the transit peptide-excluded OsAOC exists as a homotrimer. In order to model oligomers of OsAOC, an OsAOC monomer was manually generated from the homotrimer using PyMol and then used as input to Galaxy Gemini (http://galaxy.seoklab.org/cgi-bin/submit.cgi?type=GEMINI; Lee et al., 2013), which results in modeling of dimeric or trimeric structure of OsAOC. The predicted structural similarity values from Galaxy Gemini modeling are shown in Table 1. Homotrimeric structures were predicted with AtAOC2 (2BRJ), PpAOC1 (4H6B), and dirigent protein DRR206 (4REV), but a homodimer was predicted with Diels-Alderase PyrI4 (5BU3) as a template (Figure 9). Using the intensive mode of Phyre^2^ (http://www.sbg.bio.ic.ac.uk/phyre2/), a model was generated for monomeric OsAOC(I), OsAOC(II), and OsAOC(III), including presequences and TP, as indicated (Figures 3A, S6). This OsAOC structural model suggests that the TP forms an α-helix that is well-separated from the core β-barrel motif.

**Table 1:** Prediction of OsAOC homo-oligomer structures.

**No**	Template^*^	**Oligomeric state**	**Structure similarity**^†^			
			**OsAOC^#^**	**OsAOC(I)^†^**	**OsAOC(II)^†^**	**OsAOC(III)^†^**
1	2BRJ	Trimeric	0.9834	0.9133	0.8461	0.7966
2	4H6B	Trimeric	0.9670	0.8982	0.8315	0.7828
3	4REV	Trimeric	0.6398	0.6092	0.5805	0.5500
4	5BU3	Dimeric	0.6050	0.5719	0.5319	0.5577

* *PDB accession code: 2BRJ (AtAOC2), 4H6B (PpAOC1), 4REV (Dirigent protein DRR206) and 5BU3 (Diels-Alderase PyrI4). Structure similarity is measured by TM (Template-based oligomer modeling)-align as described in Lee et al. (2013). It ranges from 0 (totally different) to 1 (identical)*.

# *Monomer subunit was generated by PyMol (N-terminal TP was automatically removed by SWISS-Model)*.

† *Monomer subunit was generated using the intensive mode of Phyre^2^ (Figure S6)*.

## Discussion

### Splicing of TP

Previous studies indicate that AOCs are localized to the chloroplast via a chloroplast targeting peptide. Consistent with this, confocal fluorescence microscopy showed that OsAOC is localized to the chloroplast in rice leaves (Figure 2B). Chloroplast proteins are synthesized in the cytosol as intact precursor polypeptides, where they interact with molecular chaperones HSP70, HSP90, and 14-3-3 proteins. The molecular chaperones prevent protein mis-folding and/or aggregation (Soll and Schleiff, 2004). The OsAOC presequence/TP (Figure 3B) appears to be removed by metal ion-dependent cleavage, because the splicing of presequence and/or TP was prevented by EDTA (Figure 3B). A similar reaction occurs in the OsAOS1-OsAOC fusion protein (Figure 8B). Self-cleavage of precursor AOC has been reported previously (Kong et al., 2009). Phyre^2^ predicted that the relatively hydrophobic TP in OsAOC(III) forms an α-helix that is well-separated from the core β-barrel (Figure S6B). We speculate that auto-cleavage occurs at the junction between this α-helix and the core β-barrel.

Indeed, protease specificity prediction server (https://prosper.erc.monash.edu.au) predicted three cleavage sites of matrix metallopeptidase-2 in the recombinant OsAOCs, including the position between S and L that is located at third residue from the TP cleavage site. The cleavage site located in the boundary region between a hydrophobic α-helix of TP and β-barrel core structure of OsAOC may provide an easy to access for a metallopeptidase to the cleavage sites.

### Oligomeric state

Previous crystallographic studies report a trimeric structure for AOC (Hofmann et al., 2006; Neumann et al., 2012). Consistent with this, structural analysis presented here, including native gel electrophoresis and gel filtration in the absence or presence of protein cross-linking reagent, demonstrate that OsAOC exists as a mixture of multimers in solution (Figures 5, S3, S4). The homodimer appears to be a preferred conformation for OsAOC. The OsAOC homodimer was detected by Western blot after extensive dilution, suggesting that it reflects a specific intramolecular interaction between two OsAOC monomers. This is consistent with the observation that OsAOC homodimers persist after partial denaturation in 0.4% SDS at room temperature (Figure 6). The population distribution of OsAOC oligomers was dependent on the temperature. The OsAOC dimer is the predominant form at room temperature under partially-denaturing conditions. Consistent with this, OsAOC is predicted to have a core β-barrel, and β-sheet proteins are reported to be relatively resistant to SDS-induced kinetic instability and protein unfolding (Manning and Colon, 2004; Nielsen et al., 2007). Using Diels-Alderase protein as a template, bioinformatics modeling predicted OsAOC to be a dimer (Table 1, Figure 9). Furthermore, a structural search based on the β-barrel structure of Diels-Aderase (PyrI^4^) found that the PyrI^4^ and AOC (4H6A) are structural homologs (Zheng et al., 2016). It is interesting that both enzymes catalyze pericyclic reactions: a [4 + 2] cycloaddition is catalyzed by Diel-Alderase and 4π electrocyclization is catalyzed by AOC. In addition, AOC can be classified as a dirigent protein, because of its characteristic β-barrel structure and its stereospecificity (Pickel et al., 2012). Our previous studies indicated that rice allene oxide synthase (OsAOS1) is in the dynamic equilibrium among diverse oligomeric structures and its subunit structure strongly depends on the presence of detergents *in vitro* (Yoeun et al., 2015). These data suggest that oligomerization of OsAOS1 could be regulated *in vivo* through protein-lipid interactions in or near the membrane. This is consistent with other studies suggesting that membrane-protein interactions involving AOS facilitate transfer of highly unstable allene oxide intermediates for the cyclization into cyclopentanone derivatives in the oxylipin biosynthetic pathway.

### Substrate channeling

The AOC gene family in *A. thaliana* includes four functional genes, AtAOC1, AtAOC2, AtAOC3 and AtAOC4, and heteromerization among these isozymes has been proposed as a mechanism of regulating AOC activity *in vivo* (Stenzel et al., 2012) and *in vitro* (Otto et al., 2016). Here, we propose that multimers and/or heteromultimers of OsAOS1 and OsAOC may facilitate interactions between the two enzymes and influence their capacity for substrate channeling during catalysis. As mentioned above, this would protect the 12,13-EOT intermediate, and prevent its premature dissolution, by effectively coupling catalysis in the active sites of OsAOC and OsAOS1. We previously reported a substrate channeling effect, when OsAOC and OsAOS1 were immobilized on nanoporous rice husk silica (Le et al., 2017). Data presented here also demonstrate a hyperbolic dependence of OsAOC reaction yield on the molar ratio of OsAOC to OsAOS1, and this is consistent with the proposed substrate channeling (Figure 7A). Western blot analysis of cross-linked multimers suggest the possible formation of a 2:1 heterotrimer (MW ≈100 kDa) comprised of one OsAOC dimer and one OsAOS1 monomer (Figure 7B), although we can't completely exclude the possibility that the 100 kDa species is tetrameric OsAOC or dimeric OsAOS1. Dimeric OsAOC appears to be the predominant oligomeric form of OsAOC in solution, and in association with OsAOS1, even though OsAOC is reported to form homotrimers during crystallization (Hofmann et al., 2006; Neumann et al., 2012). Unlike *A. thaliana*, rice cells express one isoform of AOC from a single AOC gene (Dhakarey et al., 2017). Therefore, multimers that include more than one enzyme isoform can form in *A. thaliana* but not in rice. As mentioned above, we propose that dimeric OsAOC interacts with monomeric OsAOS1 to form a heterotrimer [(OsAOC)_2_OsAOS1], and that this heterotrimer is required for efficient substrate channeling during catalysis.

### AOC as a biologically analogous nazarov cyclase

**Figure 10:**
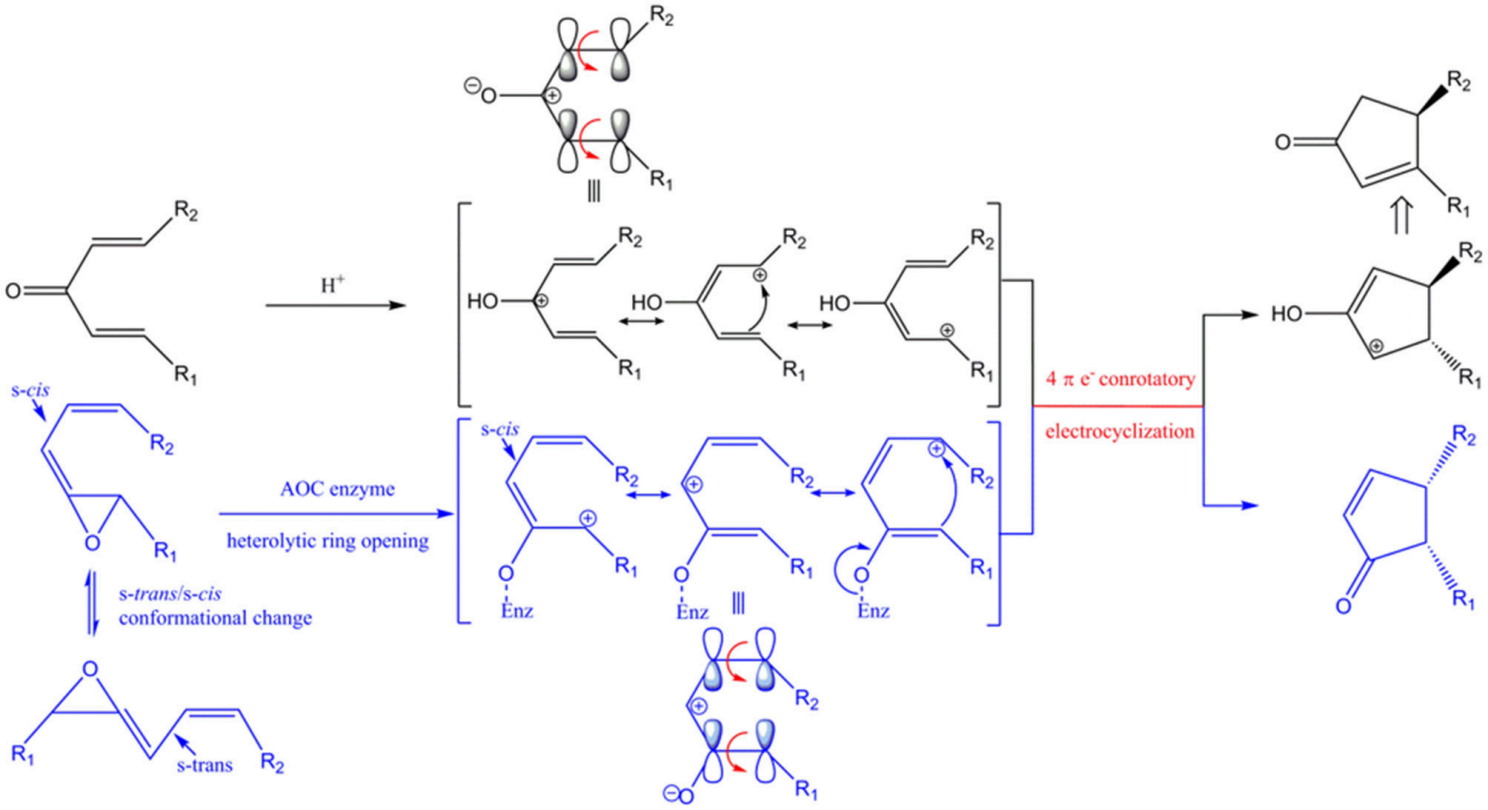
Conceptual similarities in mechanism and stereochemical control of the Nazarov cyclization (black) and the reaction catalyzed by AOC (blue). Enz represents enzyme-mediated stabilization of the oxyanion during the AOC reaction. The corresponding oxyanion in the zwitter ionic transition state is stabilized by Lewis acid (represented by H^+^). HOMO (highest occupied molecular orbital) is shown above the corresponding resonance form of the pentadienyl cation. Red arrows represent conrotatory electrocyclization. R_1_ = C_5_H_9_, R_2_ = C_7_H_14_COOH in AOC reaction.

AOC catalyzes the conversion of the allene oxide into the α,β-unsaturated cyclopetenone with stereospecific cyclization. This reaction is conceptually similar, with similar mechanism and stereo-specificity, as the Nazarov reaction (Figures 10, S1).

The unstable allene oxide readily undergoes heterolytic cleavage after the *s-trans/s-cis* conformational change, which results in a zwitter ionic transition state and the oxyanion is stabilized by specific amino acid side chains in AOC (Hofmann and Pollmann, 2008). In the analogous Nazarov reaction, the zwitter ionic transition state is generated from divinyl ketone and the oxyanion is stabilized by a Lewis acid instead of being stabilized by amino acid side-chain interactions. The stereochemistry is determined by the shared principle of peri-electrocyclization; thus, conrotatory cyclization of a 4π electron system by AOC yields *cis*-cyclopentenone, while the Nazarov reaction generates a *trans*-cyclopentenyl cation, which is subsequently converted to the cyclopenteneone by proton transfer and tautomerization. The isomeric configuration of R1 and R2 is an important determinant of the stereochemistry of the cyclized product. As far as we know, this is the first report of enzyme-catalyzed cyclization as a biological analog of Nazarov cyclization in chemistry.

## Author contributions

SY designed the experiments, analyzed data, and wrote the manuscript. KC contributed critical reading and revision of the manuscript. OH contributed scientific advice and corrected the manuscript. All authors have read and approved the manuscript.

### Conflict of interest statement

The authors declare that the research was conducted in the absence of any commercial or financial relationships that could be construed as a potential conflict of interest.

## References

[B1] Agrawal G. K. Jwa N.-S. Agrawal S. K. Tamogami S. Iwahashi H. Rakwal R. (2003). Cloning of novel rice allene oxide cyclase (OsAOC): mRNA expression and comparative analysis with allene oxide synthase (OsAOS) gene provides insight into the transcriptional regulation of octadecanoid pathway biosynthetic genes in rice. Plant Sci. 164, 979–992. 10.1016/S0168-9452(03)00082-7

[B2] Agrawal G. K. Tamogami S. Han O. Iwahashi H. Rakwal R. (2004). Rice octadecanoid pathway. Biochem. Biophys. Res. Commun. 317, 1–15. 10.1016/j.bbrc.2004.03.020 15047141

[B3] Biasini M. Bienert S. Waterhouse A. Arnold K. Studer G. Schmidt T.. (2014). SWISS-MODEL: modelling protein tertiary and quaternary structure using evolutionary information. Nucleic Acids Res. 42, W252–W258. 10.1093/nar/gku340 24782522 PMC4086089

[B4] Blée E. (2002). Impact of phyto-oxylipins in plant defense. Trends Plant Sci. 7, 315–322. 10.1016/S1360-1385(02)02290-2 12119169

[B5] Bollag D. M. Edelstein S. J. Rozycki M. D. (1996). Protein Methods. New York, NY: Wiley-Liss.

[B6] De León I. P. Hamberg M. Castresana C. (2015). Oxylipins in moss development and defense. Front. Plant Sci. 6:483 10.3389/fpls.2015.00483 26191067 PMC4490225

[B7] Dhakarey R. Raorane M. L. Treumann A. Peethambaran P. K. Schendel R. R. Sahi V. P.. (2017). Physiological and proteomic analysis of the rice mutant cpm2 suggests a negative regulatory role of jasmonic acid in drought tolerance. Front. Plant Sci. 8:1903. 10.3389/fpls.2017.01903 29250082 PMC5715382

[B8] Gleason C. Leelarasamee N. Meldau D. Feussner I. (2016). OPDA has key role in regulating plant susceptibility to the root-knot *nematode meloidogyne* hapla in arabidopsis. Front. Plant Sci. 7:1565. 10.3389/fpls.2016.01565 27822219 PMC5075541

[B9] Hofmann E. Pollmann S. (2008). Molecular mechanism of enzymatic allene oxide cyclization in plants. Plant Physiol. Biochem. 46, 302–308. 10.1016/j.plaphy.2007.12.007 18272375

[B10] Hofmann E. Zerbe P. Schaller F. (2006). The crystal structure *of Arabidopsis thaliana* allene oxide cyclase: insights into the oxylipin cyclization reaction. Plant Cell 18, 3201–3217. 10.1105/tpc.106.043984 17085685 PMC1693953

[B11] Kong F. J. Li Y. Abe J. Liu B. Schaller F. Piotrowski M.. (2009). Expression of allene oxide cyclase from Pharbitis nil upon theobroxide treatment. Biosci. Biotechnol. Biochem. 73, 1007–1013. 10.1271/bbb.80780 19420721

[B12] Le T. B. Han C. S. Cho K. Han O. (2017). Covalent immobilization of oxylipin biosynthetic enzymes on nanoporous rice husk silica for production of cis (+)-12-oxophytodienoic acid. Artif. Cells Nanomed. Biotechnol. 11, 1–7. 10.1080/21691401.2017.1375939 28889752

[B13] Lee H. Park H. Ko J. Seok C. (2013). GalaxyGemini: a web server for protein homo-oligomer structure prediction based on similarity. Bioinformatics 29, 1078–1080. 10.1093/bioinformatics/btt079 23413437

[B14] Manning M. Colon W. (2004). Structural basis of protein kinetic stability: resistance to sodium dodecyl sulfate suggests a central role for rigidity and a bias toward beta-sheet structure. Biochemistry 43, 11248–11254. 10.1021/bi0491898 15366934

[B15] Mosblech A. Feussner I. Heilmann I. (2009). Oxylipins: structurally diverse metabolites from fatty acid oxidation. Plant Physiol. Biochem. 47, 511–517. 10.1016/j.plaphy.2008.12.011 19167233

[B16] Neumann P. Brodhun F. Sauer K. Herrfurth C. Hamberg M. Brinkmann J.. (2012). Crystal structures of *Physcomitrella patens* AOC1 and AOC2: insights into the enzyme mechanism and differences in substrate specificity. Plant Physiol. 160, 1251–1266. 10.1104/pp.112.205138 22987885 PMC3490582

[B17] Nielsen M. M. Andersen K. K. Westh P. Otzen D. E. (2007). Unfolding of beta-sheet proteins in SDS. Biophys. J. 92, 3674–3685. 10.1529/biophysj.106.101238 17351005 PMC1853130

[B18] Otto M. Naumann C. Brandt W. Wasternack C. Hause B. (2016). Activity regulation by heteromerization of Arabidopsis allene oxide cyclase family members. Plants 5:3. 10.3390/plants5010003 27135223 PMC4844422

[B19] Pickel B. Pfannstiel J. Steudle A. Lehmann A. Gerken U. Pleiss J.. (2012). A model of dirigent proteins derived from structural and functional similarities with allene oxide cyclase and lipocalins. FEBS J. 279, 1980–1993. 10.1111/j.1742-4658.2012.08580.x 22443713

[B20] Raviv Z. Cohen S. Reischer-Pelech D. (2013). The anti-cancer activities of jasmonates. Cancer Chemother. Pharmacol. 71, 275–285. 10.1007/s00280-012-2039-z 23196641

[B21] Savchenko T. Kolla V. A. Wang C. Q. Nasafi Z. Hicks D. R. Phadungchob B.. (2014). Functional convergence of oxylipin and abscisic acid pathways controls stomatal closure in response to drought. Plant Physiol. 164, 1151–1160. 10.1104/pp.113.234310 24429214 PMC3938610

[B22] Schaller F. Biesgen C. Mussig C. Altmann T. Weiler E. W. (2000). 12-Oxophytodienoate reductase 3 (OPR3) is the isoenzyme involved in jasmonate biosynthesis. Planta 210, 979–984. 10.1007/s004250050706 10872231

[B23] Sharma M. Laxmi A. (2016). Jasmonates: emerging players in controlling temperature stress tolerance. Front. Plant Sci. 6:1129. 10.3389/fpls.2015.01129 26779205 PMC4701901

[B24] Soll J. Schleiff E. (2004). Protein import into chloroplasts. Nat. Rev. Mol. Cell Biol. 5, 198–208. 10.1038/nrm1333 14991000

[B25] Stenzel I. Otto M. Delker C. Kirmse N. Schmidt D. Miersch O.. (2012). ALLENE OXIDE CYCLASE (AOC) gene family members of *Arabidopsis thaliana*: tissue- and organ-specific promoter activities and *in vivo* heteromerization. J. Exp. Bot. 63, 6125–6138. 10.1093/jxb/ers261 23028017 PMC3481204

[B26] Stumpe M. Gobel C. Faltin B. Beike A. K. Hause B. Himmelsbach K.. (2010). The moss *Physcomitrella* patens contains cyclopentenones but no jasmonates: mutations in allene oxide cyclase lead to reduced fertility and altered sporophyte morphology. New Phytol. 188, 740–749. 10.1111/j.1469-8137.2010.03406.x 20704658

[B27] Taki N. Sasaki-Sekimoto Y. Obayashi T. Kikuta A. Kobayashi K. Ainai T.. (2005). 12-oxo-phytodienoic acid triggers expression of a distinct set of genes and plays a role in wound-induced gene expression in Arabidopsis. Plant Physiol. 139, 1268–1283. 10.1104/pp.105.067058 16258017 PMC1283764

[B28] Tius M. A. (2014). Allene ether Nazarov cyclization. Chem. Soc. Rev. 43, 2979–3002. 10.1039/C3CS60333D 24196585

[B29] Wasternack C. (2007). Jasmonates: an update on biosynthesis, signal transduction and action in plant stress response, growth and development. Ann. Bot. 100, 681–697. 10.1093/aob/mcm079 17513307 PMC2749622

[B30] Yamamoto Y. Ohshika J. Takahashi T. Ishizaki K. Kohchi T. Matusuura H.. (2015). Functional analysis of allene oxide cyclase, MpAOC, in the liverwort *Marchantia polymorpha*. Phytochemistry 116, 48–56. 10.1016/j.phytochem.2015.03.008 25892411

[B31] Yoeun S. Kim J. I. Han O. (2015). Cellular localization and detergent dependent oligomerization of rice allene oxide synthase-1. J. Plant Res. 128, 201–209. 10.1007/s10265-014-0670-y 25326901

[B32] Yoeun S. Rakwal R. Han O. (2013). Dual positional substrate specificity of rice allene oxide synthase-1: insight into mechanism of inhibition by type II ligand imidazole. BMB Rep. 46, 151–156. 10.5483/BMBRep.2013.46.3.117 23527858 PMC4133873

[B33] Zheng Q. Guo Y. Yang L. Zhao Z. Wu Z. Zhang H.. (2016). Enzyme-dependent [4 + 2] cycloaddition depends on lid-like interaction of the n-terminal sequence with the catalytic core in PyrI4. Cell Chem. Biol. 23, 352–360. 10.1016/j.chembiol.2016.01.005 26877021

[B34] Ziegler J. Hamberg M. Miersch O. Parthier B. (1997). Purification and characterization of allene oxide cyclase from dry corn seeds. Plant Physiol. 114, 565–573. 10.1104/pp.114.2.565 12223729 PMC158337

