# Supplementary material for: Structural Evidence for the Substrate Channeling of Rice Allene Oxide Cyclase in Biologically Analogous Nazarov Reaction
Source: Front Chem. 2018 Oct 30;6:500. doi: 10.3389/fchem.2018.00500 (PMC6218421; doi:10.3389/fchem.2018.00500)
Supplement: Supplementary file 1 [file Data_Sheet_1.zip › Data Sheet 1.PDF]

SUPPLEMENTARY MATERIALS

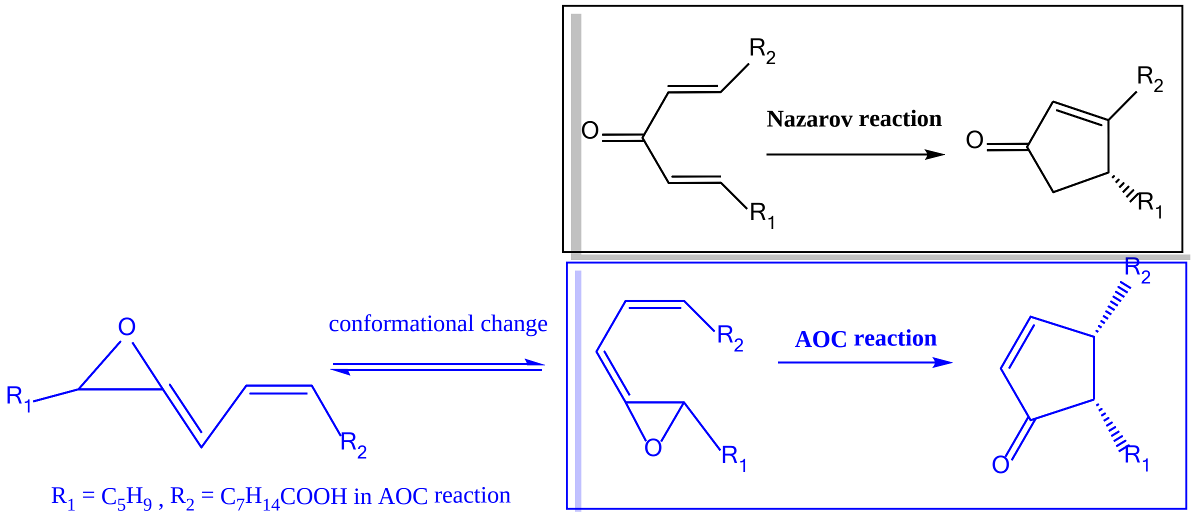

Figure S1. Formation of cyclopentenone in Nazarov (black) and AOC (blue) reactions. R_1 = C_5H_9, R_2 = $C_7H_{14}COOH$ in AOC reaction.

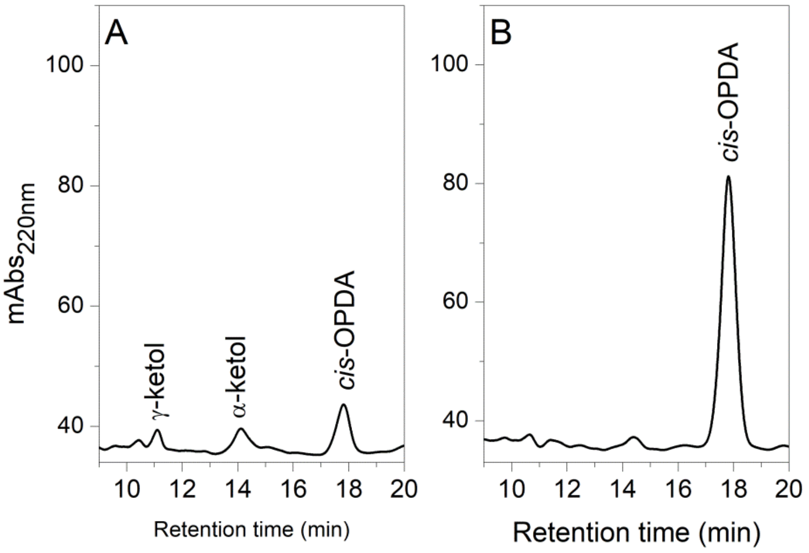

Figure S2. RP-HPLC analysis of OsAOC(I) reaction products. The reaction mixtures were injected directly onto reverse phase HPLC. **(A)** Non cyclization products of allene oxide, 12,13-EOT. The allene oxide was non-enzymatically converted into ketols and a racemic mixture of *cis*(±)-OPDA in the absence of OsAOC. **(B)** Enzymatic cyclization product of OsAOC. Formation of *cis*-OPDA increased remarkably in the presence of OsAOC.

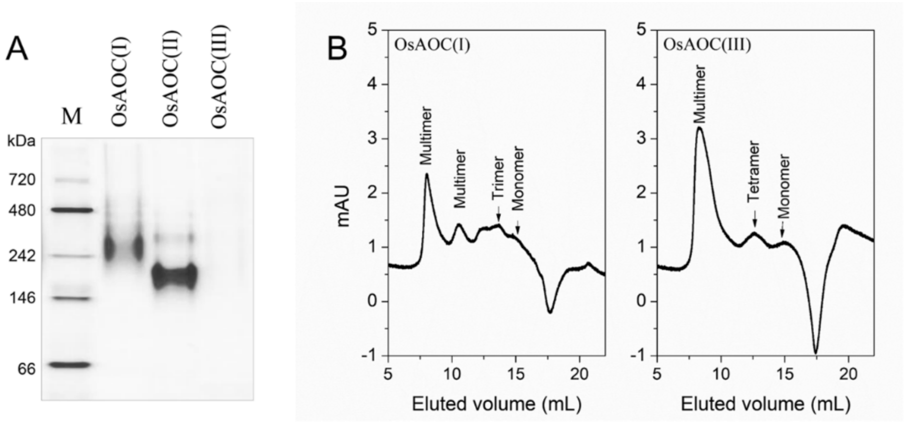

Figure S3. Analysis of oligomeric structures of OsAOC **(A)** Native PAGE Analysis. The recombinant proteins (~4 µg) were loaded into a native gel composed of 5% stacking and the 6–15% gradient separating gels. The full-length OsAOC(III) could did not migrate into the gel. (**B)** Gel filtration analysis. Molecular weight (MW) standards are void (blue dextran), β-amylase (200 kDa), BSA (66 kDa), ovalumin (44 kDa), carbonic anhydrogenase (29 kDa), and cytocrom C (12.4 kDa). The molecular size was determined from a plot of log molecular size (x) versus $K_{av}(y) = (Ve - Vo)/(Vt - Vo)$ and the data were fitted by linear regression where the coefficient of determination, $r^2 = 0.94307$, to the equation: $y = -0.49254\ x + 3.26545$.

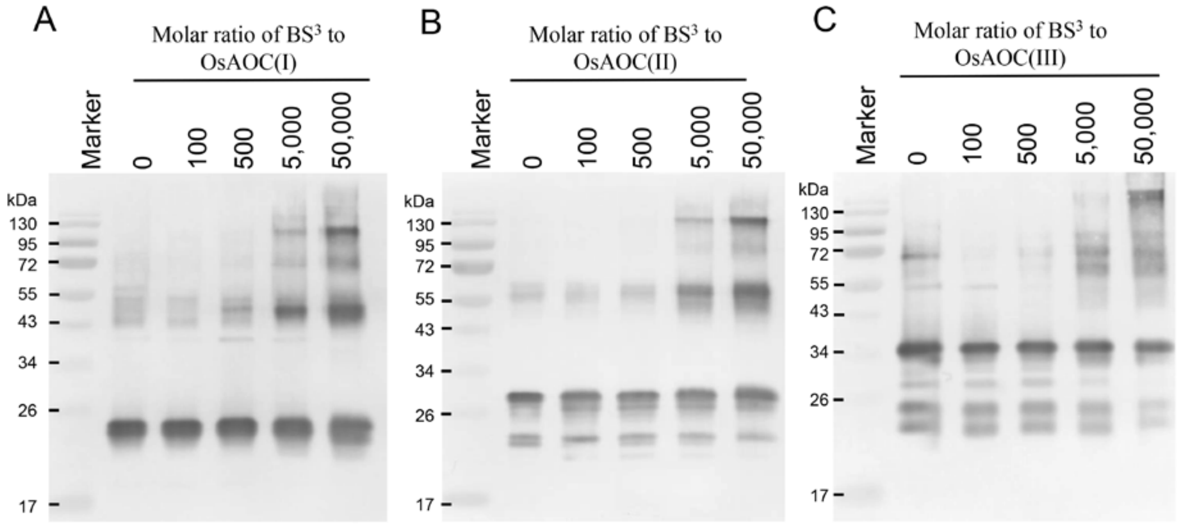

Figure S4. Western blot analysis of the cross-linked OsAOC. **(A)** OsAOC(I), **(B)** OsAOC(II), **(C)** OsAOC(III). The 100-fold diluted proteins were cross-linked with BS^3 as described in Figure 5. Reaction mixtures containing ≈ 13 ng OsAOC variant were analyzed by 15% SDS-PAGE.

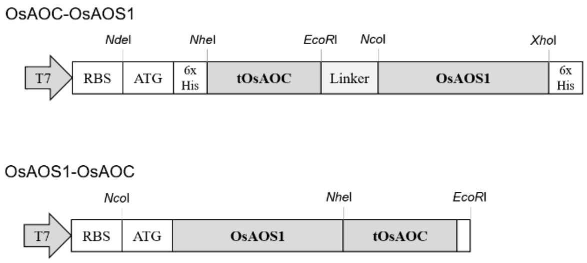

Figure S5. Plasmid constructs for OsAOC/OsAOS1 fusion proteins. OsAOC-OsAOS1: OsAOC lies N-terminal to OsAOS1. OsAOS1-OsAOC: OsAOS1 lies N-terminal to OsAOC.

A

```
OsAOC(I)     MRGSHHHHHHGM------------------------------------------------    12
OsAOC(II)    MRGSHHHHHHGMASMTGGQQMGRDLYDDDDKDPSSRSAAGTM------------------    42
OsAOC(III)   MRGSHHHHHHGMASMTGGQQMGRDLYDDDDKDPSSRSAAGTMAAAAPSRVSVRAAAPGQT    60

OsAOC(I)     ------------------------------ASLFSPKPATPKDARPAKVQEMFVYEINER    42
OsAOC(II)    ------------------------------ASLFSPKPATPKDARPAKVQEMFVYEINER    72
OsAOC(III)   GGFAKIRPQVVVAAAARSAGVSGRRARSVRASLFSPKPATPKDARPAKVQEMFVYEINER   120

OsAOC(I)     DRESPAYLRLSAKQTENALGDLVPFTNKLYSGSLDKRLGISAGICILIQHVPERNGDRYE   102
OsAOC(II)    DRESPAYLRLSAKQTENALGDLVPFTNKLYSGSLDKRLGISAGICILIQHVPERNGDRYE   132
OsAOC(III)   DRESPAYLRLSAKQTENALGDLVPFTNKLYSGSLDKRLGISAGICILIQHVPERNGDRYE   180

OsAOC(I)     AIYSFYFGDYGHISVQGPYLTYEESYLAVTGGSGVFEGAYGQVKLNQIVFPFKIFYTFYL   162
OsAOC(II)    AIYSFYFGDYGHISVQGPYLTYEESYLAVTGGSGVFEGAYGQVKLNQIVFPFKIFYTFYL   192
OsAOC(III)   AIYSFYFGDYGHISVQGPYLTYEESYLAVTGGSGVFEGAYGQVKLNQIVFPFKIFYTFYL   240

OsAOC(I)     KGIPDLPRELLCTPVPPSPTVEPTPAAKATEPHACLNNFTN   203
OsAOC(II)    KGIPDLPRELLCTPVPPSPTVEPTPAAKATEPHACLNNFTN   233
OsAOC(III)   KGIPDLPRELLCTPVPPSPTVEPTPAAKATEPHACLNNFTN   281
```

B

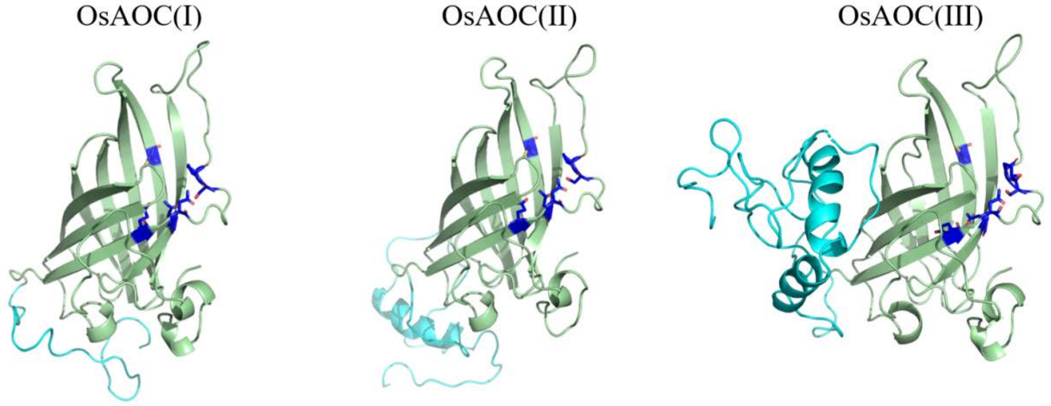

Figure S6. Structural modeling of protomeric OsAOC variants. **(A)** Sequences of N-terminal region of OsAOC variants. **(B)** Structures of protomers of OsAOC variants. Amino acid sequences of OsAOC(I), OsAOC(II), OSAOC(III) were used as input for intensive mode of Phyre^2 (http://www.sbg.bio.ic.ac.uk/phyre2/) and protomer structure was generated. Light blue (cyan) indicates the part removed in SWISS-Model-generated structure of AtAOC2 (see text). Blue denotes OsAOC active site amino acid residues that play critical roles during catalysis.
